# Membrane Association Allosterically Regulates Phospholipase A_2_ Enzymes and Their Specificity

**DOI:** 10.1021/acs.accounts.2c00497

**Published:** 2022-10-31

**Authors:** Varnavas D. Mouchlis, Edward A. Dennis

**Affiliations:** Department of Chemistry and Biochemistry and Department of Pharmacology, School of Medicine, University of California, San Diego, La Jolla, California 92093-0601 United States

## Abstract

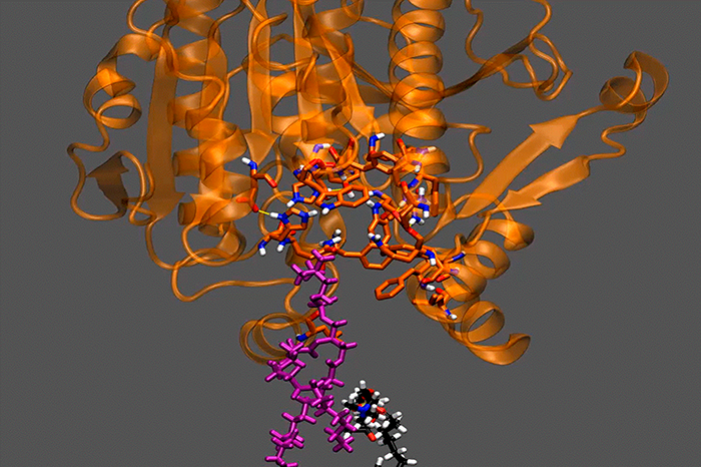

Water-soluble proteins as well as membrane-bound proteins associate with membrane surfaces and bind specific lipid molecules in specific sites on the protein. Membrane surfaces include the traditional bilayer membranes of cells and subcellular organelles formed by phospholipids. Monolayer membranes include the outer monolayer phospholipid surface of intracellular lipid droplets of triglycerides and various lipoproteins including HDL, LDL, VLDL, and chylomicrons. These lipoproteins circulate in our blood and lymph systems and contain triglycerides, cholesterol, cholesterol esters, and proteins in their interior, and these are sometimes interspersed on their surfaces. Similar lipid–water interfaces also occur in mixed micelles of phospholipids and bile acids in our digestive system, which may also include internalized triglycerides and cholesterol esters. Diacyl phospholipids constitute the defining molecules of biological membranes. Phospholipase A_1_ (PLA_1_) hydrolyzes phospholipid acyl chains at the *sn*-1 position of membrane phospholipids, phospholipase A_2_ (PLA_2_) hydrolyzes acyl chains at the *sn*-2 position, phospholipase C (PLC) hydrolyzes the glycerol–phosphodiester bond, and phospholipase D (PLD) hydrolyzes the polar group–phosphodiester bond. Of the phospholipases, the PLA_2_s have been the most well studied at the mechanistic level. The PLA_2_ superfamily consists of 16 groups and numerous subgroups, and each is generally described as one of 6 types. The most well studied of the PLA_2_s include extensive genetic and mutational studies, complete lipidomics specificity characterization, and crystallographic structures. This Account will focus principally on results from deuterium exchange mass spectrometric (DXMS) studies of PLA_2_ interactions with membranes and extensive molecular dynamics (MD) simulations of their interactions with membranes and specific phospholipids bound in their catalytic and allosteric sites. These enzymes either are membrane-bound or are water-soluble and associate with membranes before extracting their phospholipid substrate molecule into their active site to carry out their enzymatic hydrolytic reaction. We present evidence that when a PLA_2_ associates with a membrane, the membrane association can result in a conformational change in the enzyme whereby the membrane association with an allosteric site on the enzyme stabilizes the enzyme in an active conformation on the membrane. We sometimes refer to this transition from a “closed” conformation in aqueous solution to an “open” conformation when associated with a membrane. The enzyme can then extract a single phospholipid substrate into its active site, and catalysis occurs. We have also employed DXMS and MD simulations to characterize how PLA_2_s interact with specific inhibitors that could lead to potential therapeutics. The PLA_2_s constitute a paradigm for how membranes interact allosterically with proteins, causing conformational changes and activation of the proteins to enable them to extract and bind a specific phospholipid from a membrane for catalysis, which is probably generalizable to intracellular and extracellular transport and phospholipid exchange processes as well as other specific biological functions. We will focus on the four main types of PLA_2_, namely, the secreted (sPLA_2_), cytosolic (cPLA_2_), calcium-independent (iPLA_2_), and lipoprotein-associated PLA_2_ (Lp-PLA_2_) also known as platelet-activating factor acetyl hydrolase (PAF-AH). Studies on a well-studied specific example of each of the four major types of the PLA_2_ superfamily demonstrate clearly that protein subsites can show precise specificity for one of the phospholipid hydrophobic acyl chains, often the one at the *sn*-2 position, including exquisite sensitivity to the number and position of double bonds.

## Key References

- MouchlisV. D.; BucherD.; McCammonJ. A.; DennisE. A.Membranes serve as allosteric activators of phospholipase A_2_, enabling it to extract, bind, and hydrolyze phospholipid substrates. Proc. Natl. Acad. Sci. U S A2015, 112, E516–E52510.1073/pnas.142465111225624474PMC4330758.^1^ First demonstration that membrane association causes an allosteric change in the enzyme conformation of phospholipase A_2_ when it is associated with a membrane.
- MouchlisV. D.; ChenY.; McCammonJ. A.; DennisE. A.Membrane Allostery and Unique Hydrophobic Sites Promote Enzyme Substrate Specificity. J. Am. Chem. Soc.2018, 140, 3285–329110.1021/jacs.7b1204529342349PMC5846079.^2^ Broad lipidomics analysis of phospholipase A_2_ specificity is first determined and correlated with molecular dynamics simulations to explain the binding specificity at the active site of c-, i-, and s-phospholipase A_2_s.
- HayashiD.; MouchlisV. D.; DennisE. A.Omega-3 versus Omega-6 fatty acid availability is controlled by hydrophobic site geometries of phospholipase A2s. J. Lipid Res.2021, 62, 10011310.1016/j.jlr.2021.10011334474084PMC8551542.^3^ Detailed comparison of c-, i-, and s-phospholipase A_2_’s specificity and differing hydrophobic binding site geometries for the carbon chain length and the degree of unsaturation in the fatty acid in the sn-2 position of omega-3 and omega-6 fatty acids in phospholipids.
- MouchlisV. D.; HayashiD.; VasquezA. M.; CaoJ.; McCammonJ. A.; DennisE. A.. Lipoprotein-associated phospholipase A_2_: A paradigm for allosteric regulation by membranes. Proc. Natl. Acad. Sci. U.S.A2022, 119, e210295311810.1073/pnas.210295311834996868PMC8764669.^4^ Demonstration of the conformational change that results from the association of lipoprotein-associated phospholipase A_2_ with a membrane and the correlation of lipidomics-determined specificity with sn-2 hydrophobic subsite geometry.

## Introduction to the Phospholipase A_2_ Superfamily

The most well characterized phospholipase A_2_ (PLA_2_) enzymes are water-soluble peripheral enzymes that associate with the surface of membranes to extract and bind their phospholipid substrates.^5,6^ However, there are some PLA_2_s that are membrane-bound but they have been less well studied, and this Account will focus on the most well studied water-soluble PLA_2_s. To access its substrate, a PLA_2_ enzyme needs to overcome the lipid/water interface barrier.^5,6^ As a result, PLA_2_ enzymes evolved to contain unique interfacial surfaces that contain amphipathic helices facilitating the association process. The association of a PLA_2_ with the membrane causes a conformational change in the protein at an allosteric site distinct from its active site.^1^ In addition, each enzyme has a unique active site that is either highly specific for a fatty acid at the *sn*-2 position or more permissive depending on the cellular processes with which the enzyme is involved.^2^ In fact, the PLA_2_s have evolved very specific geometries at their specific hydrophobic site, which binds the fatty acyl group at the *sn*-2 position to result in highly specific substrate specificity for their biological function as determined by lipidomics analysis.^3,4^

Since PLA_2_ enzymes catalyze hydrolysis at the *sn*-2 position, the type of fatty acid at the *sn*-1 position is not expected to play a significant role in their activity. However, in some cases it seems that the ester bond at the *sn*-1 position is preferred versus the ether bond of the platelet activating factor (PAF) or vinyl ethers of plasmalogens.^7^ Hydrogen–deuterium exchange mass spectrometry (HDX-MS) is a powerful biophysical technique that has been used extensively to study the interactions of PLA_2_ enzymes with membranes and vesicles.^8^ Molecular dynamics simulations guided by HDX-MS results have proven to be very useful in understanding the association and inhibition mechanisms of PLA_2_ enzymes and in generating enzyme–inhibitor complexes for designing new inhibitors with improved properties.^9^

The PLA_2_ superfamily consists of 16 groups and many subgroups of structurally and functionally diverse enzymes.^10^ The six types of PLA_2_ enzymes include the secreted (sPLA_2_), cytosolic (cPLA_2_), calcium-independent (iPLA_2_), and lipoprotein-associated PLA_2_ (Lp-PLA_2_) also known as platelet-activating factor acetylhydrolase (PAF-AH), lysosomal PLA_2_, and adipose-PLA (AdPLA).^6,10^ Numerous important functions are attributed to the PLA_2_ superfamily as summarized in Table 1.^6^ Chief among them is the important role of PLA_2_ in generating inflammatory mediators, especially cPLA_2_, for which the group IVA cPLA_2_ shows dramatic specificity for the release of free eicosatetraenoic acid known as arachidonic acid (AA) from the *sn*-2 position of membrane phospholipids. Arachidonic acid is an omega-6 fatty acid which is generally considered to be pro-inflammatory.^11^

**Table 1:** Functions of the Human PLA_2_ Superfamily^a^

type	major groups	major functions
sPLA_2_	groups IB, IIA, IID, IIE, V, X, XIIA	digestion, antibacterial, antiviral, tumorigenesis, pro-inflammatory, anti-inflammatory
cPLA_2_	group IVA (cPLA_2_α)	pro-inflammatory
iPLA_2_	group VIA (iPLA_2_β)	remodeling of *sn*-2 fatty acids, insulin sensitivity, mitochondrial function (Barth syndrome)
LpPLA_2_	group VIIA (PAF-AH)	anti-inflammatory, pro-inflammatory
LPLA_2_		lysosomal phospholipid hydrolysis
AdPLA		phospholipid hydrolysis in adipose tissue

a Adapted with permission from ref (6). Copyright 2011 American Chemical Society.

The AA is further converted by cyclooxygenases (COX) to various tissue-specific prostaglandins that play potent cell signaling and pro-inflammatory roles by binding to specific G-protein coupled receptors (GPCRs) and activating various peroxisome-proliferator activated receptors (PPARS).^11^ In certain tissues, the AA is converted enzymatically to various HETEs and leukotrienes by various lipoxygenases, which activate other GPCRs and PPARS. We will discuss the specificity of the various types/groups of PLA_2_ at the molecular level herein. Of special current interest is determining which PLA_2_ types (and specific groups and subgroups) are responsible for releasing the pro-inflammatory mediator arachidonic acid versus those PLA_2_s that favor the omega-3 fish oil-derived fatty acids eicosapentaenoic acid (EPA) and docosahexaenoic acid (DHA), which are often precursors of anti-inflammatory or pro-resolution lipid mediators.^11^ We recently determined that while GIVA cPLA_2_ prefers the omega-6 pro-inflammatory AA as a substrate in the *sn*-2 position of its phospholipid substrates, omega-3 fatty acid docosahexaenoic acid (DHA), which is generally considered to be anti-inflammatory or pro-resolution, is preferred by group V sPLA_2_.^3^ The functional pro- and anti-inflammatory activities and other functions in specific tissues and cells of each of the sPLA_2_s are reviewed elsewhere, including pro-inflammatory properties of GIV cPLA_2_ and anti-inflammatory properties of GIID sPLA_2_.^12^

In this Account, we will summarize several studies that reveal the association mechanism of group IVA cytosolic (cPLA_2_), group VIA calcium-independent (iPLA_2_), group V secreted (sPLA_2_), and group VIIA lipoprotein-associated phospholipase A_2_ (GVIIA Lp-PLA_2_), also known as platelet-activating factor (PAF) acetylhydrolase (PAF-AH) with membranes. Our studies revealed that these enzymes are allosterically regulated by membranes through their interfacial surface. Substrate specificity studies showed that each enzyme contains a distinct binding pocket for the *sn*-2 fatty acid with amino acid side chains that make it specific for a type of fatty acid. Inhibitor studies suggested that these binding sites could be used to achieve inhibition selectivity.

## Membranes Induce Allosteric Conformational Changes in Associated Proteins

**Figure 1:**
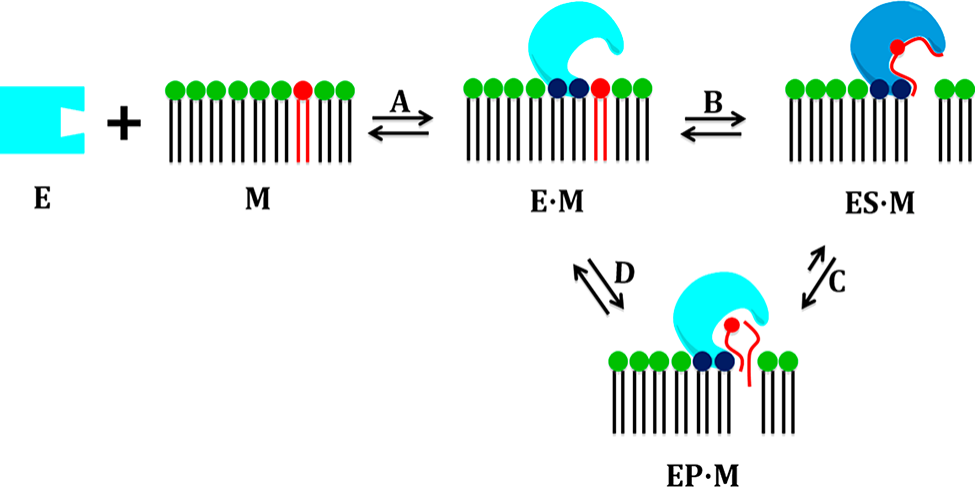
Catalytic cycle of PLA_2_ enzymes. The association of each enzyme with the membrane causes an allosteric conformational change that enables it to extract and bind a phospholipid molecule. Reproduced with permission from ref (2). Copyright 2018 American Chemical Society.

PLA_2_ enzymes need to associate with an aggregated form of their phospholipid substrate including bilayer membranes and phospholipid vesicles as well as phospholipid monolayers surrounding lipoproteins, lipid droplets, liposomes, and mixed micelles (the subject of a previous Account)^13^ in order to extract and bind a single phospholipid molecule.^8^ “Surface-dilution kinetics” was successfully employed to interpret the kinetics of PLA_2_s.^14,15^ It proposed that the enzymes first undergo surface association to the membranes depending on the bulk concentration of enzyme and membranes. In a second distinct step, the enzyme pulls a phospholipid substrate into its catalytic site, which depends on the substrate phospholipid concentration in the two-dimensional interface. Each enzyme contains an interfacial surface, which has unique topology and function, and it facilitates the association with the membrane or any other form of lipid aggregate. A combination of HDX-MS with molecular dynamics simulations has proven to be very successful in understanding the association mechanism of the pure, recombinant human form of specific group/subgroups GIVA cPLA_2_, GV sPLA_2_, GVIA iPLA_2_, and GVIIA Lp-PLA_2_. For simplicity, in this Account, we will refer to these enzymes as cPLA_2_, sPLA_2_, iPLA_2_, and Lp-PLA_2_. These studies showed that PLA_2_ enzymes exist in at least three conformations including the “closed” conformation in water (E), the “open” membrane associated “unbound” conformation (E·M), and the “open” membrane-associated “bound” conformation (ES·M) after the extraction of the phospholipid molecule in the active site (Figure 1).^2^ In this section, we will discuss the mechanism of the association of PLA_2_s with the membrane through their interfacial surface and its associated interactions.

**Figure 2:**
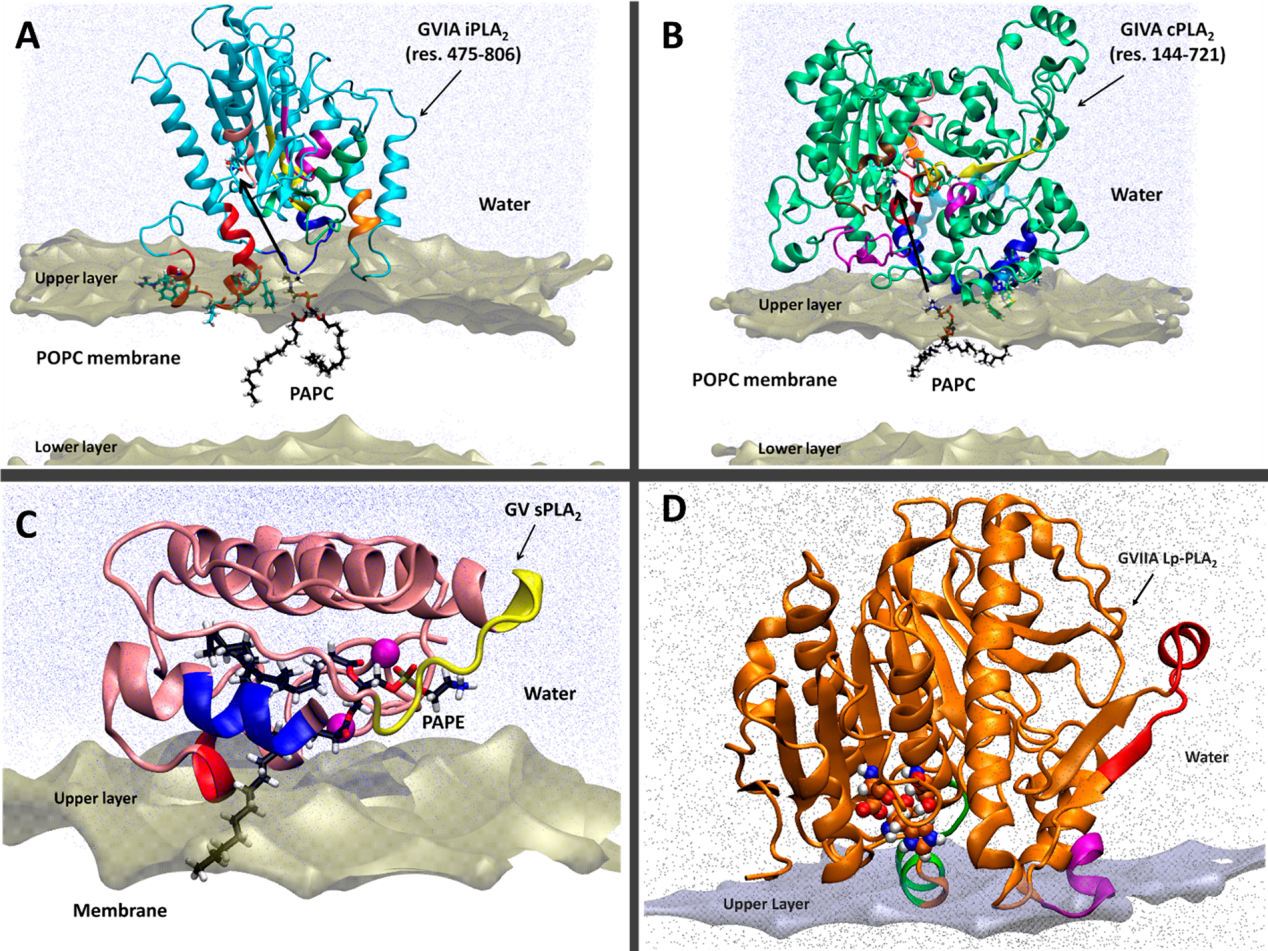
Association of each PLA_2_ enzyme with the membrane bilayer through the interfacial surface. (A) iPLA_2_, (B) cPLA_2_, (C) sPLA_2_, and (D) Lp-PLA_2_. Adapted with permission from refs (2) and (4). Copyright 2018 American Chemical Society and copyright 2022 National Academy of Sciences, respectively.

HDX-MS studies on the iPLA_2_ enzyme in the presence of phospholipid vesicles led to the identification of the peptide regions of the enzyme that interact with the membrane.^16^ Among them, peptide region 708–730 showed a significant decrease in deuteration levels in the presence of phospholipid vesicles. Coarse-grained and atomistic molecular dynamics were successful in understanding the association of iPLA_2_ with a lipid bilayer.^17^ The proposed model suggested that the peptide region 708–730, which is close to the active site, penetrates the lipid/water interface and forms an amphipathic helix with its hydrophilic residues (Arg710 and Lys719) interacting with the headgroups of the fatty acids and its hydrophobic residues (Pro711, Pro714, Trp715, Leu717, Val721, and Phe722) interacting with the acyl chains of the fatty acids (Figure 2A).^1^ It is worth mentioning that homology modeling and later the crystal structure revealed an ankyrin repeat region along with the catalytic domain of iPLA_2_ that did not show any interactions with the membrane bilayer.^1,18^

Similar HDX-MS studies were performed on cPLA_2_ to identify the peptide regions of the interfacial surface that interact with the membrane. The cPLA_2_ structure contains a C2 domain along with the catalytic domain that was thought to assist the association of the enzyme with the membrane through its calcium binding site.^19,20^ The HDX-MS studies showed several peptide regions on the C2 domain and the catalytic domain exhibiting decreases in deuteration levels in the presence of phospholipid vesicles.^21^ According to our model, two regions of the C2 domain (residues 35–39 and 96–98) penetrate the membrane approximately 15 Å, bringing along the catalytic domain to near the membrane surface. Residues 268–279 and 466–470 are two peptide regions of the catalytic domain that are close to the surface of the membrane containing some hydrophobic residues (Trp464 and Met468) interacting with the fatty acid tails of the phospholipids and some hydrophilic residues (Lys273, Lys274, and Arg467) interacting with the headgroups of the phospholipids (Figure 2B).^1^

As with the iPLA_2_ and cPLA_2_ enzymes, sPLA_2_ also exhibits an increase in activity due to interfacial activation in the presence of a membrane or any other form of lipid aggregates.^22,23^ HDX-MS studies on group IA sPLA_2_ showed that peptide regions 3–8, 18–21, and 56–64 exhibited a significant decrease in the “on-exchange” rates in the presence of dimyristoylphosphatidylcholine phospholipid vesicles. These peptide regions have an amphipathic nature containing hydrophobic residues that penetrate the membrane and hydrophilic residues that interact with the headgroups on the surface of the membrane.^24^ Along with GIA sPLA_2_, we were also interested in the human GV sPLA_2_, and this later enzyme was used in our detailed substrate specificity studies. Since there is no crystal structure available for GV sPLA_2_, a homology model was generated based on the crystal structure of GIIA sPLA_2_ because these two enzymes have 47% identity and 61% homology. In general, there is high homology between sPLA_2_ enzymes, and that allowed us to use the HDX-MS peptide regions to generate a structural model for GV sPLA_2_ docked on the surface of the membrane (Figure 2C). This model was subjected to extensive molecular dynamics simulations in the presence of various phospholipid substrates. These studies allowed us to determine the structural features that contribute to substrate selectivity, and they will be discussed in detail in the next section of this Account.

Lp-PLA_2_ is also membrane-associated and undergoes interfacial activation like c-, i-, and sPLA_2_ enzymes. According to our HDX-MS studies, peptide regions 114–120 and 360–368 exhibited decreases in on-exchange rates in the presence of phospholipid vesicles (Figure 2D). These two regions constitute two amphipathic helices that play a significant role in protein–membrane binding because their properties allow them to overcome the water–lipid energy barrier.^25−27^ An enzyme-membrane model was generated based on the HDX-MS data and was subjected to extensive molecular dynamics simulations to study the interfacial activation mechanism of Lp-PLA_2_.^4^ The simulations showed that in the presence of the membrane, the volume of the Lp-PLA_2_ active site was increased from ∼900 to 2000 Å^3^ due to a conformational change caused by the region 100–130. When the open form of the enzyme was placed in water, a conformational change in the same region caused the enzyme to adopt a closed form.^4^ Similar conformational changes were confirmed for c-, i-, and sPLA_2_.^2^

## Specific Phospholipase A_2_ Binding Sites Extract and Bind a Specific Phospholipid Molecule and Determine the Phospholipid Substrate Specificity

Phospholipids are highly flexible molecules and are often very challenging to cocrystallize with PLA_2_ enzymes. Thus, there are no available crystal structures to help understand the binding mode and interactions of phospholipids with the active site of PLA_2_ enzymes. Computational methods such as molecular docking and induced-fit docking could not handle the high flexibility of phospholipids.^28^ Atomistic molecular dynamics at a microsecond scale have proven successful in developing enzyme–substrate models. These models help understand the binding mode and interactions of phospholipids with each PLA_2_ active site and explain each enzyme’s specificity for various types of phospholipid molecules. Simulations were carried out for c-, i-, s-, and Lp-PLA_2_ enzymes with phospholipids containing various types of fatty acids at the *sn-*2 position.^1,2,4^ Arachidonic (20:4), linoleic (18:2), myristic (14:0), and azelaoyl (9:0, COOH) esterified at the *sn*-2 position of membrane phospholipids will be discussed in this Account because they are the optimum substrates for cPLA_2_, i and sPLA_2_, and Lp-PLA_2_, respectively.

**Figure 3:**
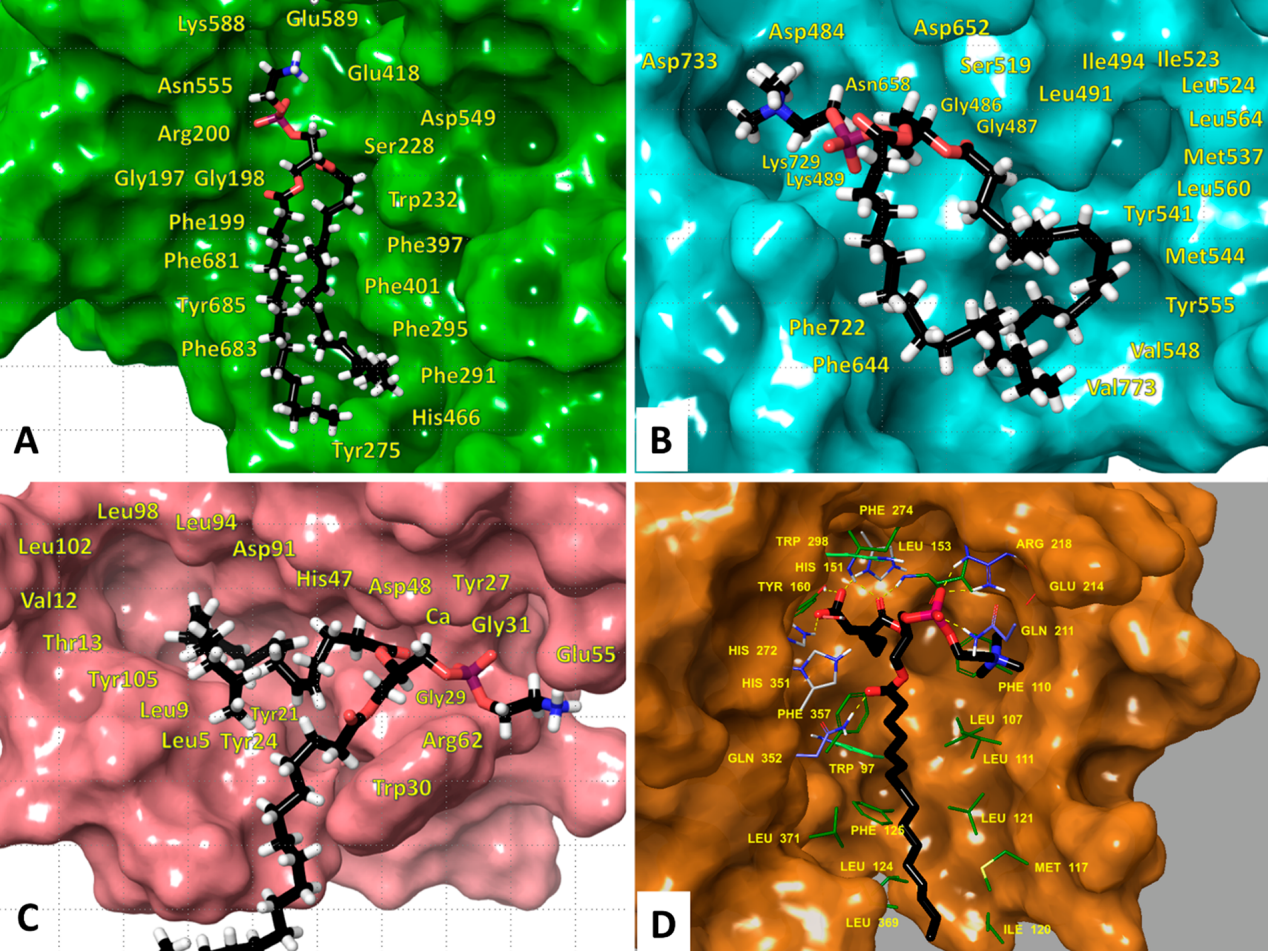
Molecular dynamics simulations revealed the structural features that contribute to substrate specificity. (A) cPLA_2_, (B) iPLA_2_, (C) sPLA_2_, and (D) Lp-PLA_2_. Adapted with permission from refs (2) and (4). Copyright 2018 American Chemical Society and copyright 2022 National Academy of Sciences, respectively.

The active site of cPLA_2_ contains a deep channel containing a large hydrophobic binding pocket that accommodates the two acyl chains of a phospholipid molecule. Enzymatic specificity studies on cPLA_2_ showed that this enzyme is highly specific for arachidonic acid at the *sn*-2 position. The simulations showed that cPLA_2_ contains a hydrophobic pocket that is rich with aromatic residues including Phe199, Phe291, Phe295, Trp232, Phe397, Phe401, Phe681, Phe683, and Tyr685 that act as a fingerprint recognizing and interacting with the double bonds of arachidonic acid (20:4) through π–π stacking (Figure 3A).^2^ iPLA_2_ exhibited optimum activity for phospholipids containing linoleic (18:2) and myristic (14:0) acid at the *sn*-2 position. The simulations showed that iPLA_2_ contains two hydrophobic binding pockets for the *sn*-2 fatty acid tail: one that could accommodate myristic (14:0) (not shown) and a second that could accommodate linoleic (18:2) (shown) (Figure 3B). The first hydrophobic pocket contains residues such as Leu491, Ile494, Ile523, Leu524, Leu564, Met537, and Leu560, and it has a suitable site to accommodate the acyl chain of the myristic (14:0) acid. The second hydrophobic pocket contains residues such as Tyr541, Tyr544, and Phe644, which interact with the double bonds of the linoleic (18:2) fatty acid.^2^

sPLA_2_ is a small 14 kDa enzyme which exhibits specificity toward linoleic (18:2) and myristic (14:0) acid at the *sn*-2 position, similarly to iPLA_2_.^2,9^ The simulations showed that sPLA_2_ also contains two hydrophobic pockets that could accommodate either the linoleic (18:2) or the myristic (14:0) fatty acid tail at the *sn*-2 position (Figure 3C). The first pocket contains residues such as Val12, Leu102, Leu98, and Leu94, and it accommodates the shorter saturated myristic (14:0) acyl chain. The second pocket contains residues such as Tyr21, Tyr24, and Tyr105 that interact with the double bonds of the linoleic (18:2) acyl chain through π–π stacking interactions. Lp-PLA_2_ exhibits greater enzymatic activity toward oxidized-PC (phosphocholine) substrates or PAF (platelet activating factor) compared to normal phospholipid substrates.^4,5^ The simulations showed that Gln352 forms a stronger hydrogen bond with the oxygen atom of the *sn*-1 carbonyl group of the PC analogues versus the ether oxygen of the PAF analogues (Figure 3D). A smaller semihydrophilic pocket was also identified in the Lp-PLA_2_ active site containing residuesTyr160, His151, and His272 forming hydrogen bonds with the carbonyl group of the oxidized phospholipids.

## Therapeutic Targeting through Inhibitor Interactions with Highly Evolved Phospholipid Hydrophobic Acyl Chain Subsites

Phospholipid substrate specificity is tightly connected to the hydrophobic pockets of the PLA_2_ active site. Thus, inhibitor selectivity is also driven by the hydrophobic pockets of the PLA_2_ active site. Molecular dynamics simulations guided by HDX-MS were extensively used by our group to understand inhibitor interactions of PLA_2_ enzymes, especially for c- and iPLA_2_ since no cocrystallized structures are available.^29,30^ Fluoroketone inhibitors are a class of potent and selective inhibitors developed for iPLA_2_ that are a clear example of how substrate specificity could be translated into inhibitor selectivity.^31−33^ The fluoroketone group of these inhibitors interacts with the “oxyanion hole”, which consists of two glycine residues in both c- and iPLA_2_ and is in close proximity to the catalytic serine which is also common in both enzymes. Despite the common interaction pattern of the fluoroketone moiety, our structure–activity relationships revealed that fluoroketone compounds with specific hydrophobic tails could be highly selective toward iPLA_2_.

**Figure 4:**
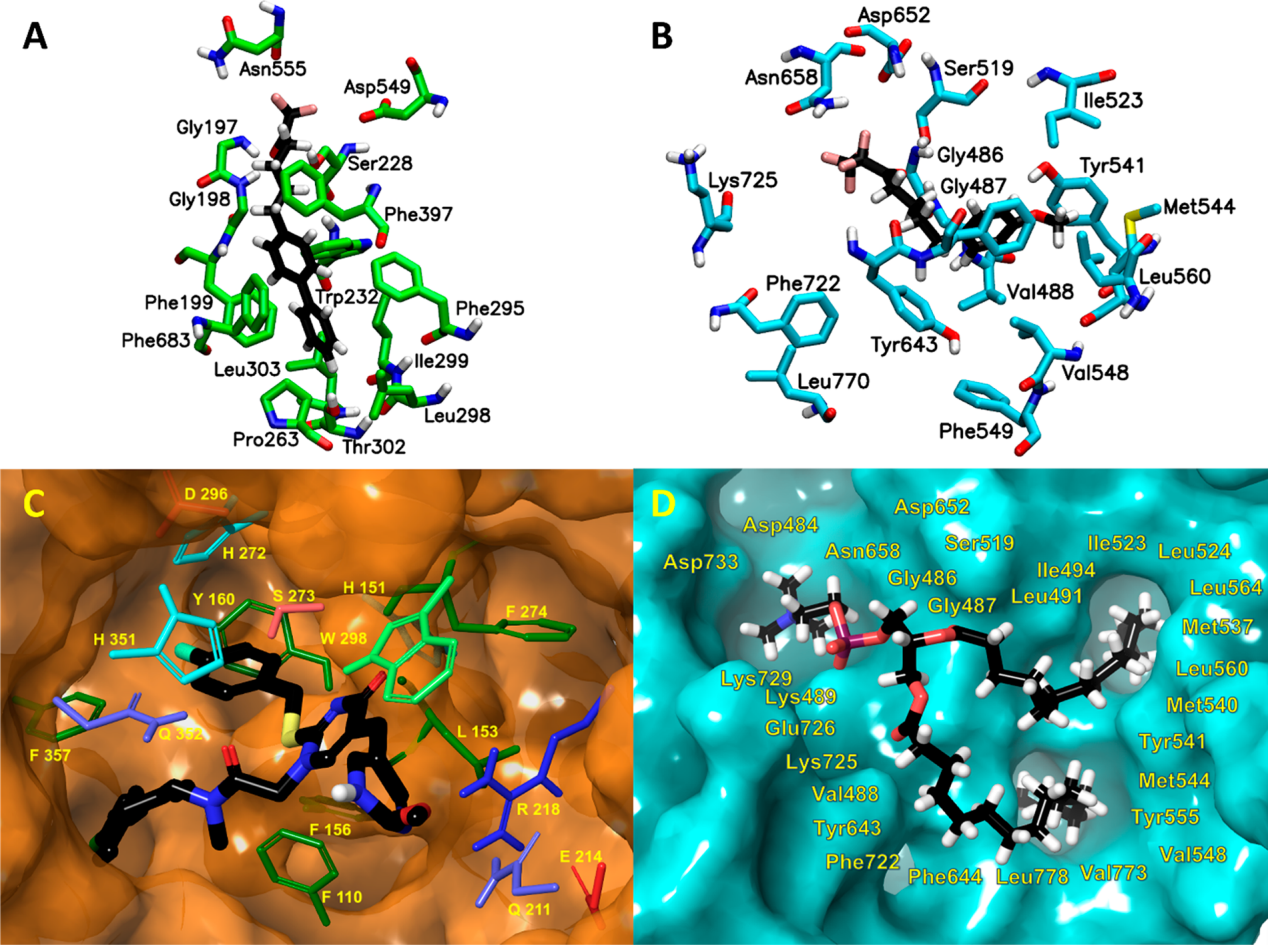
Specificity of each phospholipase A_2_ hydrophobic binding site for specific substrates and inhibitors. Binding mode of fluoroketone inhibitors in the active sites of (A) cPLA_2_ and (B) iPLA_2_. (C) Binding mode of a potent specific inhibitor in the active site of Lp-PLA_2_. (D) Binding mode of a phospholipid containing myristic acid at the *sn*-2 position in the active site of iPLA_2_. Adapted with permission from refs (2), (4), and (31). Copyright 2018 American Chemical Society, copyright 2022 National Academy of Sciences, and copyright 2016 American Chemical Society, respectively.

Based on the simulations of phospholipid substrates, it was found that cPLA_2_ contains a deep hydrophobic pocket that accommodates the *sn*-2 arachidonic tail. This pocket contains many aromatic residues that interact with the double bonds of the arachidonic tail. There are two factors that govern tight binding in this hydrophobic pocket: the size of the ligand and its aromaticity. Fluoroketone compounds that contain longer aromatic hydrophobic tails tend to be potent inhibitors for cPLA_2_ (Figure 4A). Such compounds contain large enough tails to complement the hydrophobic pocket of cPLA_2_ and at the same time form π–π stacking with aromatic residues such as Phe199, Phe291, Phe295, Trp232, Phe397, Phe401, Phe681, Phe683, and Tyr685. Larger compounds such as oxoamides containing long aliphatic or aromatic tails and are also potent and selective inhibitors of cPLA_2_.^34−36^

The hydrophobic part of the iPLA_2_ active site consist of two pockets: one that contains aromatic residues and accommodates the *sn*-2 linoleic chain and one that contains aliphatic residues and accommodates the *sn*-2 myristic chain.^2^ Fluoroketone compounds that contain shorter aromatic tails tend to be potent and selective inhibitors for iPLA_2_. The aromatic pocket of iPLA_2_ has a significantly smaller volume than the one in cPLA_2_ because the *sn*-2 site in iPLA_2_ is much more specific for linoleic acid at the *sn*-2 position. In addition, the active site of iPLA_2_ is more flexible, and the inhibitor locks the enzyme in the closed conformation in which the volume of the hydrophobic pocket is significantly smaller (Figure 4B).^31^ Interestingly, very potent inhibitors of Lp-PLA_2_ contain aromatic residues that specifically associate with aromatic residues in the protein active site (Figure 4C).^4^ It is also worth noting that fluoroketone compounds containing only short aliphatic tails are also potent and selective inhibitors for iPLA_2_. Molecular dynamics simulations combined with HDX-MS studies showed that the aliphatic tail binds to the smaller aliphatic pocket of iPLA_2_ that accommodates the *sn*-2 myristic chain in myristoyl-containing phospholipids (Figure 4D).^32^

Our recently developed lipidomic assay for c-, i-, s-, and Lp-PLA_2_ enzymes, using existing potent inhibitors for each enzyme, provided a semihigh throughput setup for performing virtual and experimental screening to identify new chemical matter for PLA_2_ inhibition.^37^ Having in mind the above structural characteristics of the c- and iPLA_2_ hydrophobic pockets, a virtual screening workflow was developed, including clustering analysis, docking in multiple enzyme conformations, and interaction criteria to select compounds from libraries to be tested *in vitro*.^38^ This work led to the identification of novel micromolar hits that are currently under development. sPLA_2_ also contains an aromatic and an aliphatic hydrophobic pocket, and it is inhibited by indole compounds that contain short aliphatic and aromatic chains.^37^ Lp-PLA_2_ contains a large hydrophobic pocket that accommodates the *sn*-1 acyl chain and a smaller amphipathic pocket that accommodates short-chain oxidized *sn*-2 chains. Compounds that contain a long chain that occupies the *sn*-1 pocket and a shorter chain that binds the *sn*-2 chain are potent inhibitors for this enzyme (Figure 4C).^4^

Members of the phospholipase A_2_ superfamily are responsible for numerous important biological functions, but they have also been implicated in numerous deleterious physiological effects on lipid metabolism with significant disease implications. This includes primary roles for cPLA_2_ in arthritis and a multitude of inflammatory diseases, sPLA_2_ and Lp-PLA_2_ in various cardiovascular diseases, and iPLA_2_ in diabetes and a variety of neurological diseases. Over the years, numerous inhibitors with excellent potency and specificity for a particular PLA_2_ were synthesized, and the pharmaceutical industry has pursued the development of several as potential therapeutic moieties. This has resulted in many clinical trials, including several large phase 3 trials. Kokotos and co-workers^39,40^ have recently reviewed the patent and clinical trials literature and report that, surprisingly, to date no approved drug has emerged. Perhaps the insights into the importance of the *sn*-2 site for binding potency and specificity discussed in this Account will provide new avenues of focus for the development of successful therapeutic agents.

## Conclusions

Receptors, phospholipid transfer and exchange proteins, and enzymes often exhibit remarkable specificity for specific phospholipids in each of their *sn*-1 acyl chain, *sn*-2 acyl chain, and polar group subsites. It is likely that membranes induce allosteric conformational changes in proteins associated with bilayer membranes both intra- and extracellularly as well as when associated with monolayers surrounding lipoproteins and lipid droplets. These interactions occur at the lipid–water interface of membranes. The interaction of PLA_2_ with bilayer membranes and with its specific phospholipid substrate in its active site and especially the specificity for the *sn*-2 fatty acyl chain serve as a general paradigm for the allosteric regulation of proteins by membranes

A recent review^41^ focused on the early development of the detailed kinetic concepts, experimental approaches, and results of molecular dynamics simulations including links to the resulting movies, which led to the concept of allosteric regulation by membranes and the demonstration of the importance of the *sn*-2 site for the specificity of PLA_2_ enzymes. In the current Account, we have presented an expanded comprehensive general picture of allosteric regulation by membranes and the specificity of hydrophobic subsites in enzymes that act in or on membranes.

This was possible because of the recent demonstration^4^ of the allosteric regulation and specificity of Lp-PLA_2_, which complements the earlier work on the three traditional cellular PLA_2_s. Of special note is the recent results^3^ comparing the differential selectivities of c-, s-, and iPLA_2_ for phospholipids containing in the *sn*-2 position the omega-3 fatty acid EPA or DHA compared to the omega-6 fatty acid AA. In total, these experimental and computational approaches allow us to articulate the structural features of each enzyme that contribute to its substrate potency and specificity. By connecting substrate specificity with inhibitor structure–activity relationships, we can now extend the substrate specificity determinations to determine the precise factors contributing to inhibitor potency and selectivity. This information should aid in the development of a general approach to designing novel chemical moieties for PLA_2_-selective inhibition.
